# Identification of Prognostic Factors in Esophageal Cancer Using Machine Learning: A Retrospective Study Based on the SEER Database

**DOI:** 10.3390/jcm15083049

**Published:** 2026-04-16

**Authors:** Piman Pocasap, Sarinya Kongpetch, Auemduan Prawan, Karnchanok Kaimuangpak, Laddawan Senggunprai

**Affiliations:** Department of Pharmacology, Faculty of Medicine, Khon Kaen University, Khon Kaen 40002, Thailand; sarinyako@kku.ac.th (S.K.); peuamd@kku.ac.th (A.P.); karnkai@kku.ac.th (K.K.); laddas@kku.ac.th (L.S.)

**Keywords:** esophageal cancer, SEER, machine learning, prognosis, survival

## Abstract

**Background:** Esophageal cancer (EC) is an aggressive malignancy with low survival rates, making accurate prognosis critical for guiding treatment decisions. Traditional prognostic methods, while essential, often lack precision and comprehensive data insights. This study aims to apply machine learning (ML) approaches to investigate EC prognosis by identifying key factors associated with 5-year survival. **Methods:** Multiple ML algorithms—Random Forest (RF), Artificial Neural Networks (ANN), K-Nearest Neighbors (KNN), AdaBoost, and Naïve Bayes—were applied to a dataset from the SEER database. Model development included exploratory data analysis, internal validation, and 5-fold cross-validation. Traditional survival analysis methods, such as Cox regression and Kaplan–Meier (KM) analysis, were integrated to further explore relationships between key predictor and outcome variables. Additionally, time-series analysis was conducted to examine survival trends over time and identify influencing factors. **Results:** RF demonstrated the highest predictive performance among the models tested. Key prognostic factors identified included surgery, summary stage, tumor size, metastasis, AJCC M stage, and age. An exploratory analysis of temporal trends further showed changes in survival outcomes across diagnosis years. **Conclusions:** The findings highlight the potential of ML approaches to analyze prognostic patterns in EC. Integrating ML models with traditional statistical analyses helped identify key prognostic factors such as surgery, summary stage, and metastasis, while the exploratory temporal analysis provided additional context regarding survival trends over time. While promising, further external validation and addressing time-series challenges are necessary. Overall, this study demonstrates the potential of ML to support the identification of prognostic factors in EC and may contribute to more informed clinical decision-making.

## 1. Introduction

Esophageal cancer (EC) is a serious disease in which malignant cells form in the tissues of the esophagus, leading to a particularly aggressive and deadly condition. It is recognized as a significant health problem worldwide, being the eighth most commonly diagnosed cancer [1], and is notorious for its very low survival rates, with median survival ranging from 8 to 13 months depending on the stage of the disease [2]. Prognosis in cancer is critical to patient care and disease management, especially in EC, where poor outcomes underscore the importance of understanding the disease trajectory to guide treatment decisions and improve survival. Traditionally, prognosis has relied on staging, histological typing, tumor size and location, which are essential and foundational tools in the management of EC [3]. However, these methods have limitations in their precision and depth of information, highlighting the need for more advanced approaches to better predict patient outcomes and inform clinical strategies [4].

In response to the limitations of traditional methods, more comprehensive approaches, such as nomograms, image-based analyses, and machine learning (ML), have been developed for cancer prognosis. These methods may provide more individualized and data-driven assessment than conventional tools alone [5,6,7]. Several ML algorithms have shown promising predictive performance in a range of cancers, including breast, colon, and ovarian cancer [8,9,10]. In addition, SEER-based studies using ML have become increasingly common. However, despite the growing literature, there remains a need for studies in EC that not only evaluate prognostic performance across different ML algorithms, but also link these findings to clinically interpretable survival analyses. In particular, identifying prognostic patterns through ML and examining whether these findings are consistent with conventional approaches may improve the clinical interpretability of model-derived results.

The primary objective of this study was to apply ML approaches to investigate the prognosis of EC using 5-year survival as the outcome variable. To strengthen clinical interpretation, the key predictors identified by ML were further examined using conventional survival analyses. Several ML algorithms that have shown predictive utility in previous cancer survival studies were included in the analysis [11,12,13]. Model development followed a structured process, including exploratory data analysis, internal validation, and 5-fold cross-validation to reduce overfitting and assess robustness. To complement the ML approach, conventional survival analyses, including Cox regression and Kaplan–Meier (KM) analysis, were also performed to further examine the relationships between key predictors and survival outcomes [14]. In addition, an exploratory analysis of temporal trends was conducted to examine how survival in EC changed across diagnosis years. This analysis was intended to provide a descriptive overview of patterns over time and to add a broader temporal perspective to the study findings. Importantly, all data used in this study were obtained from the freely available SEER database, ensuring that the research is accessible and reproducible for further studies in this field.

## 2. Materials and Methods

### 2.1. Data Collection

Esophageal cancer data were collected from the National Cancer Institute (NCI) SEER Research Data, 17 Registries (2000–2021), using SEER*Stat software (version 8.4.3). The inclusion criteria include the patients diagnosed with primary cancer (Primary by international rules: Yes) of esophagus (ICD-O-3/WHO 2008: Esophagus) who were diagnosed between 2004 and 2015. The baseline dataset contains 43,764 records.

### 2.2. Data Selection

**Figure 1:**
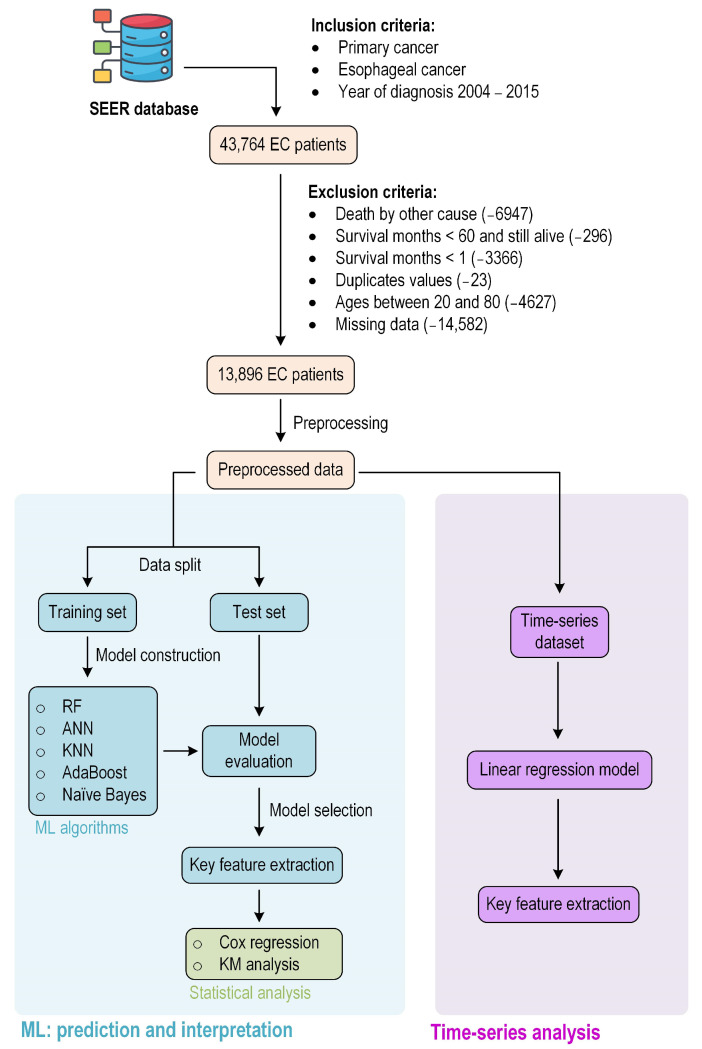
Research flowchart.

The available variables were then selected from the baseline dataset based on demographic, clinicopathological, treatment, and survival data, using R (version 4.3.1) (R Foundation for Statistical Computing, Vienna, Austria). The records with vital status “Alive” and survival months less than study cut-off point (60 months) were censored, as were cases of death attributed to other causes or unknown causes. Any missing data was removed. All data inclusion and exclusion criteria are displayed in Figure 1. The completed dataset contains 13,896 records with 26 original predictor variables.

### 2.3. Data Preprocessing

Data preprocessing was performed using Python (version 3.10.14) (Python Software Foundation, 2024). The binary outcome variable, 5-year survival, was derived from survival months variable (0: survival months < 60, and 1: survival months ≥ 60). Min–Max scaling was applied to continuous variables, while categorical variables were transformed using one-hot encoding. Feature selection was subsequently applied to the dataset. Extreme Gradient Boosting (XGBoost) was used for this step because it is a tree-based method that can effectively estimate feature importance while accommodating nonlinear relationships and demonstrating relative robustness to multicollinearity [15]. The features (variables) were then selected based on feature importance scores. Variables with importance scores above the median were retained as a simple and reproducible threshold to reduce dimensionality while preserving informative features. The preprocessed dataset contains 61 predictor variables.

### 2.4. Machine Learning Models

The preprocessed dataset was randomly split into a training set (70%) and a test set (30%). Data normalization was performed using the training set and then applied to the test set. Continuous variables were normalized using Min–Max scaling, while categorical variables were encoded using one-hot encoding. To address data imbalance, SMOTE (Synthetic Minority Over-sampling Technique) was applied exclusively to the training set, generating synthetic samples for the minority class and thereby preventing data leakage and ensuring an unbiased evaluation of model performance [16]. The proportion of the minority class increased from 21.8% before SMOTE to 50.0% after SMOTE. Five-fold cross-validation was employed during model construction to validate the consistency and robustness of the predictive models across different subsets of the data. To enable comparison across models with different learning principles, five machine-learning algorithms were selected: Random Forest (RF), Artificial Neural Network (ANN), K-Nearest Neighbors (KNN), AdaBoost, and Naive Bayes. These models were chosen to represent commonly used classification approaches with distinct underlying assumptions and analytical characteristics, thereby allowing a broader evaluation of predictive performance. RF and AdaBoost were included as ensemble-based methods suitable for structured data; ANN was selected for its capacity to capture complex nonlinear relationships; KNN was included as a nonparametric distance-based classifier; and Naive Bayes was used as a probabilistic baseline model.

### 2.5. Model Evaluations

To evaluate model performance, three classification metrics were used to provide a comprehensive comparison based on TP (true positive), TN (true negative), FP (false positive), and FN (false negative) [17]:

Accuracy: The overall correctness of the model.

$$Accuracy=\frac{TP+TN}{TP+TN+FP+FN}$$

F1 score: A harmonic mean of precision and recall.

$$F1\;Score=2\times \frac{Precision\times Recall}{Precision+Recall}$$

ROC-AUC: Area under the ROC (Receiver Operating Characteristic) curve, which plots true positive rate (TPR or Recall) against false positive rate (FPR) across different thresholds.

$$AUC=\int_{0}^{1}TPR\left(FPR\right)\;dFPR$$

where

$$Precision=\frac{TP}{TP+FP},$$

$$TPR\;\left(Recall\right)=\frac{TP}{TP+FN},$$

and

$$FPR=\frac{FP}{FP+TN}$$

Subsequently, the scores from each metric were summed, and the predictive model with the highest cumulative score was selected. Feature importance was then analyzed using a method appropriate for the selected machine learning algorithm to identify key features for further analysis.

### 2.6. Linear Regression for Exploratory Temporal Analysis

The time-series dataset was constructed from the preprocessed data based on the year of diagnosis variable, covering the period from 2004 to 2015. For continuous variables, median values were computed by grouping the data by year of diagnosis and calculating the median for each year. In contrast, categorical variables (including outcome variable) were summarized as percentages. This resulted in a dataset with 12 rows (2004–2015) and 62 columns (61 predictor variables and 1 outcome variable).

Variance thresholding was applied to the dataset to remove low variance variables, ensuring only features with sufficient variability were retained. Following this, Recursive Feature Elimination (RFE) with Ridge regression as the optimizer was employed to further reduce the number of variables. Correlation analysis was then conducted to eliminate any remaining multicollinear data. Finally, multiple linear regression was performed on the entire dataset, with 5-year survival as the outcome variable. The model’s coefficients were used to identify the key features contributing to the linear model, highlighting the most influential variables.

### 2.7. Statistical Analysis

For statistical comparison between the training and test sets, chi-square tests were employed for categorical variables, and Mann–Whitney U tests were utilized for continuous variables. Kaplan–Meier survival analysis, along with the log-rank test, and multivariate Cox proportional hazards regression analysis were performed to evaluate the relationship between key features—identified by the best-performing model—and survival months. This relationship was further substantiated by using Mann–Whitney U tests to compare survival months across different categories. All statistical analyses were conducted using Python.

## 3. Results

### 3.1. Data Exploratory Analysis

A cohort of 43,764 patients diagnosed with primary EC was identified from the SEER database for the period spanning 2004 to 2015. After applying exclusion criteria, 29,868 patients were removed, leaving a final sample size of 13,896 patients for analysis. Notably, only complete cases were included in the analysis. A total of 14,582 cases were excluded because of missing data, which may have introduced selection bias and reduced cohort representativeness. Imputation was not performed because missingness was substantial in clinically important variables and potentially non-random. The study examined demographic, clinicopathologic, and treatment-related factors. Demographic variables included race, sex, and age. Clinicopathologic data covered histopathology, tumor grade, tumor location, and metastasis status. Treatment data encompassed the use of chemotherapy, radiation, and surgery. The complete set of variables, detailed in Table 1, consists of 26 predictor variables (before encoding) and one outcome variable.

**Figure 2:**
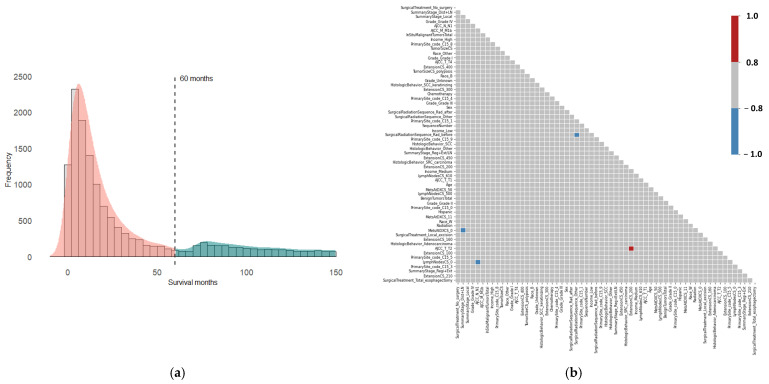
Distribution of survival months (**a**) and correlation matrix of the preprocessed dataset (**b**). The color bar represents the correlation coefficients.

Given that survival month is the outcome variable, it was prioritized for detailed examination. The distribution of survival months deviates from normality, with a median survival of 15 months. Notably, only 22% of patients survived beyond 60 months, indicating a class imbalance in the outcome variable (Figure 2a). The 5-year survival variable, a binary outcome, was derived from the survival months variable using a cutoff point of 60 months. For the predictor variables, the data underwent preprocessing, including normalization and encoding, resulting in 126 predictors. To refine the dataset, feature selection techniques were applied, reducing the number of predictor variables to 61.

**Figure 3:**
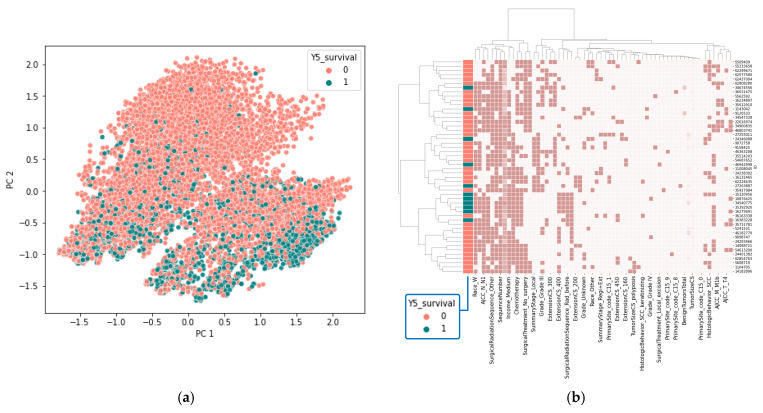
PCA scatter plot (**a**) and hierarchical cluster analysis (**b**) for assessing 5-year survival data clustering using the preprocessed dataset. In the hierarchical cluster analysis, rows represent 50 randomly selected patient records as representative samples, while columns represent variables.

Since multicollinearity can impact the performance of machine learning models, a correlation matrix was constructed for the predictor variables to assess the degree of multicollinearity (Figure 2b). The analysis revealed that the preprocessed dataset contained only a few instances of multicollinearity, allowing us to retain all the data without further modifications. Principal component analysis (PCA) was then performed to explore potential data clustering. The results showed that PCA was ineffective in distinguishing between the 5-year survival groups (0 and 1), as the data points in the PCA plot largely overlapped (Figure 3a). Likewise, hierarchical cluster analysis failed to separate the groups based on 5-year survival (Figure 3b).

Our exploratory data analysis reveals that the outcome variable is imbalanced, and the dataset shows a high degree of homogeneity across predictor variables. These characteristics make it challenging for unsupervised learning methods, such as PCA and hierarchical clustering, to effectively distinguish between the groups. This situation suggests the use of more advanced approaches, such as supervised machine learning techniques, which can utilize labeled data to achieve more accurate and reliable classification.

### 3.2. ML Model Performance

The preprocessed dataset was divided into training and test sets to evaluate the performance of various supervised learning algorithms. As shown in Table 1, there were no significant differences between the training and test sets for most variables, except for a minor discrepancy in the chemotherapy variable (73% vs. 71%, *p* < 0.01). This indicates that the data in both sets are largely homogeneous. The models selected for this study included RF, ANN, KNN, AdaBoost, and Naïve Bayes. Given the imbalanced nature of the data, SMOTE was applied to the training set to address the underrepresentation of minority classes. To ensure the robustness of the model evaluations, 5-fold cross-validation was conducted on the training set.

**Figure 4:**
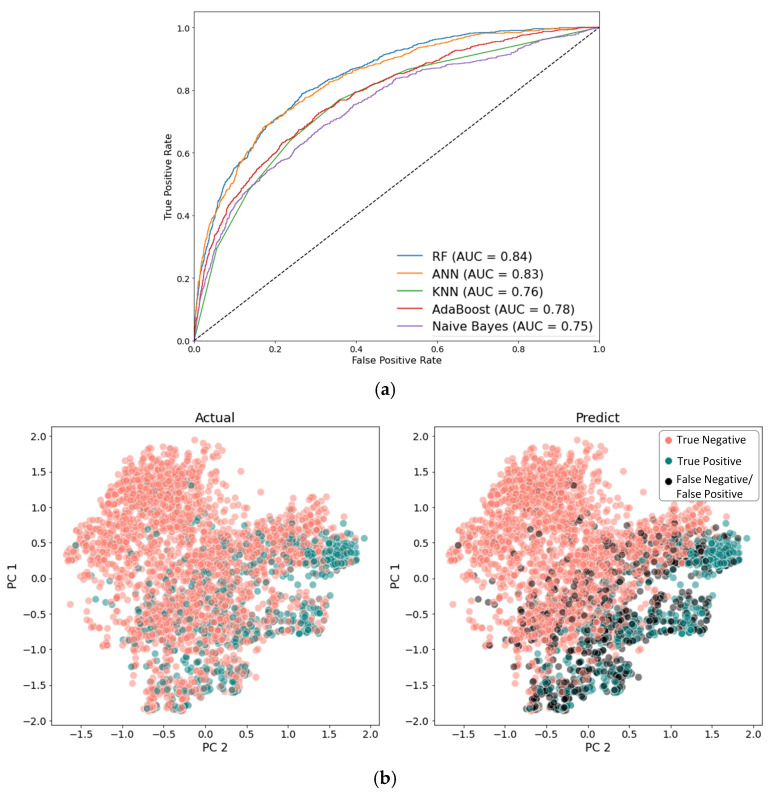
ROC-AUC curves (**a**) for model performance evaluation, and PCA scatter plot (**b**) comparing ground truth labels (actual) with RF-classified labels (predict). ROC-AUC: Area under the Receiver Operating Characteristic curve; RF: Random Forest; ANN: Artificial Neural Network; KNN: K-Nearest Neighbors.

Following the training phase, the models were validated using the test set. Due to the presence of class imbalance, evaluation metrics extended beyond simple accuracy. Metrics such as ROC-AUC and F1 score were also considered to provide a more comprehensive assessment of the models’ performance [18]. Because no single metric fully captured model performance in this imbalanced setting, a simple summed score of the evaluation metrics was used as a practical aid for overall model ranking. The ROC-AUC curves, which demonstrate the models’ ability to differentiate between classes in the test set, are presented in Figure 4a. The confusion matrices for all models are provided in the Supplementary Materials. Detailed performance metrics for all models, evaluated on the test set, are summarized in Table 2.

Among the algorithms evaluated, the RF model achieved the highest overall performance, particularly excelling in ROC-AUC and F1 scores. This indicates a strong ability to correctly classify both majority and minority classes. To further analyze the predictions, PCA was employed to visualize the distribution of predicted outcomes, as depicted in Figure 4b. Based on these results, the RF model was chosen for further analysis due to its consistent performance across multiple evaluation criteria, making it the most effective model in this study.

### 3.3. ML Model Interpretation

**Figure 5:**
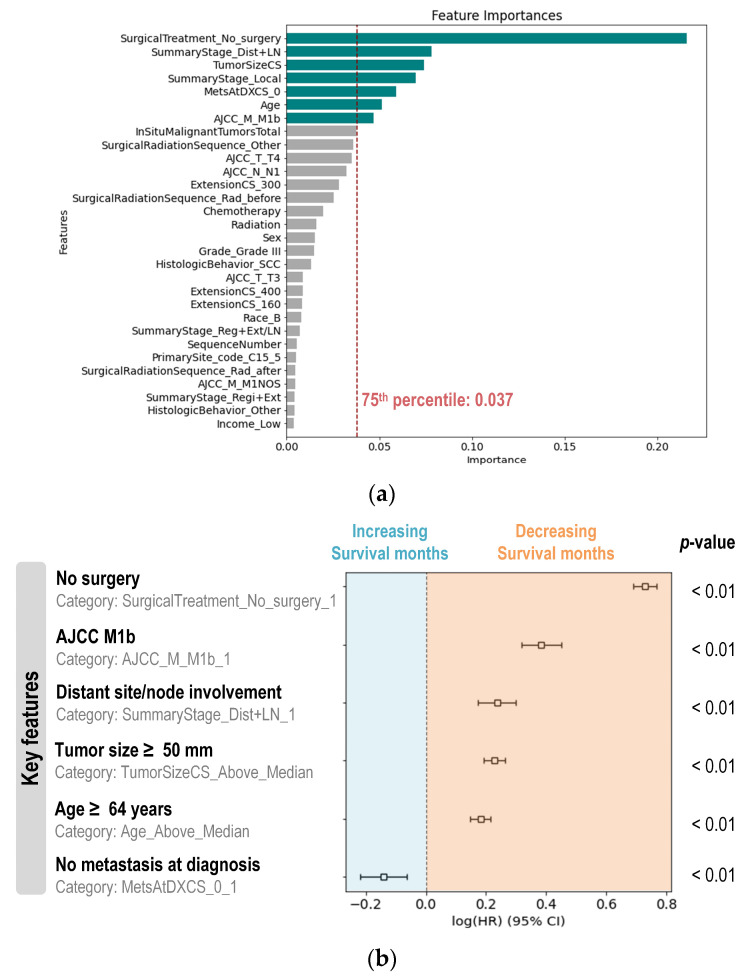
Feature importance scores (**a**) from the Random Forest (RF) model, and Cox hazard ratio plot (**b**) for key features extracted from the RF model. Features with importance scores above the 75th percentile are highlighted in teal. The Wald test was used for multivariate analysis in the Cox regression. Continuous variables, including tumor size and age, were converted to categorical variables for simpler interpretation. The reference categories for comparison include SurgicalTreatment_No_surgery_0, AJCC_M_M1b_0, SummaryStage_Dist+LN_0, TumorSizeCS_Below_Median, Age_Below_Median, and MetAtDXCS_0_0, respectively.

To interpret RF model, feature importance scores were extracted using the built-in Gini metrics, which quantify each variable’s contribution to the model’s predictions. The ranked importance scores for each variable are presented in Figure 5a. Variables with scores above 75th percentile were identified as significant contributors to data discrimination, including surgery, summary stage, tumor size, metastasis, AJCC M stage, and age. Notably, within the summary stage variable, only the “Dist+LN” category was selected for further analysis due to its higher importance score.

To explore the impact of these key variables on patient survival outcomes, a comprehensive analysis was conducted using the entire dataset. A multivariate Cox regression analysis was employed to assess the influence of these variables on survival time. The results revealed that all selected variables were statistically significant predictors of patient survival, indicating a strong association with survival outcomes (Figure 5b). This analysis provided insights into how each variable contributes to mortality risk, offering a detailed understanding of the factors affecting patient prognosis.

**Figure 6:**
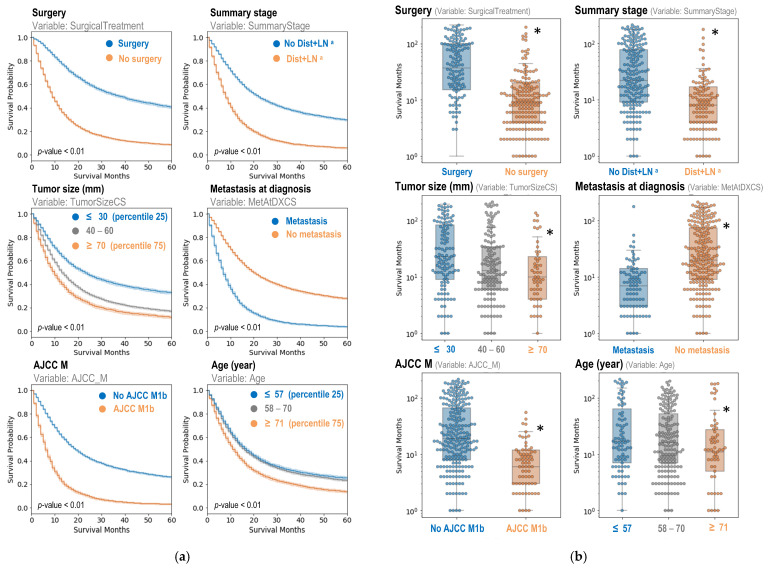
Kaplan–Meier (KM) survival plot (**a**) and box plot of survival months (**b**) for key features extracted from the Random Forest (RF) model. Continuous variables, including tumor size and age, were converted to categorical variables based on the 25th and 75th percentiles for simpler interpretation. In the KM analysis, the log-rank test was used to determine statistical significance between categories. In the box plot analysis, the Mann–Whitney U test was used for statistical comparison between categories. * *p* < 0.01 compared with the corresponding category (orange vs. blue label). ^a^ Refers to code 7 (Distant site(s)/node(s) involved).

Following the Cox regression analysis, KM plots were generated, accompanied by log-rank tests, to further investigate the effects of each variable on patient survival. The KM plots visually represented survival probabilities over time for different categories within each variable, demonstrating significant differences in survival rates (Figure 6a). These plots confirmed that the key variables have a substantial impact on patient survival, with distinct survival curves for each category.

To further substantiate these findings, survival months were plotted against each variable’s different categories, and statistical tests were conducted to assess the significance of differences (Figure 6b). The results were consistent with the conclusions drawn from the Cox regression and KM analysis, reinforcing the critical role of these variables in determining patient survival. This comprehensive analysis underscores the importance of these predictors in the context of patient outcomes, highlighting their specific influences on survival.

### 3.4. Exploratory Temporal Analysis

**Figure 7:**
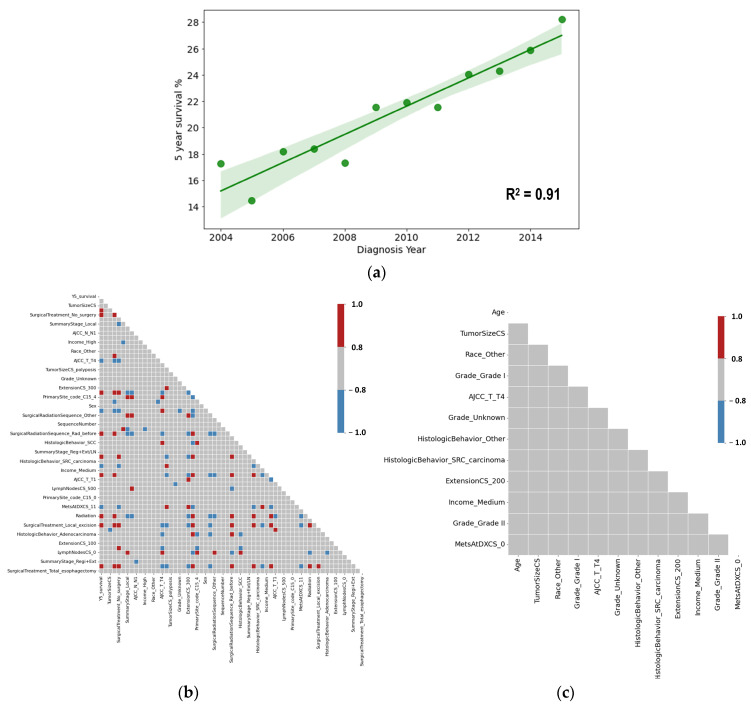
Scatter plot of diagnosis year versus 5-year survival (**a**), and correlation matrices before (**b**) and after (**c**) the feature selection process. In the scatter plot, the green line indicates a linear correlation between diagnosis year and 5-year survival, with an R^2^ of 0.91, while the shaded green band represents the 95% confidence interval. The color bars in the correlation matrices represent the correlation coefficients.

A time series dataset was constructed from the preprocessed data, resulting in 12 rows (representing diagnosis years from 2004 to 2015) and 62 columns (comprising 61 predictor variables and 1 outcome variable). The analysis began with plotting the percentage of patients achieving 5-year survival against the year of diagnosis. This plot exhibited a strong linear relationship, with an R^2^ value of 0.91, indicating a high degree of linear correlation (Figure 7a). This finding suggested a clear temporal pattern in the 5-year survival rate across diagnosis years and supported the use of multiple linear regression as an exploratory tool for examining temporal associations with the available variables.

To prepare for multiple regression analysis, an initial examination of multicollinearity among the variables was conducted using a correlation matrix (Figure 7b). The analysis revealed significant multicollinearity in the time series dataset, which could adversely affect the regression model’s reliability. To address this issue, several feature selection techniques were employed, including variance thresholding, RFE, and correlation analysis. These methods reduced the number of predictor variables from 62 to 12. After refining the dataset, the correlation matrix was re-evaluated, confirming the absence of significant multicollinearity among the selected features (Figure 7c).

**Figure 8:**
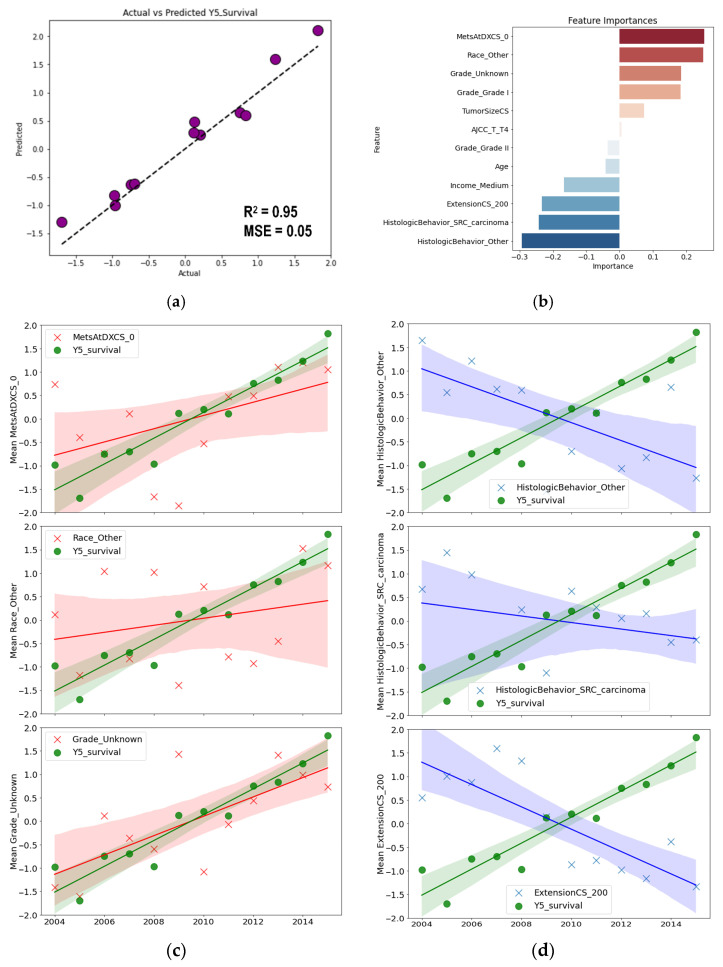
Actual vs. predicted plot (**a**) and feature importance scores (**b**) of the constructed linear regression model. The positive (**c**) and negative (**d**) relationships between selected features and 5-year survival are illustrated. The shaded band represents the 95% confidence interval. The extracted features and their interpretation are as follows: MetAtDXCS_0 (No metastasis at diagnosis), Race_Other (Races other than white and black), Grade_Unknown (Unspecified grading), HistologicBehavior_Other (Unspecified histology), HistologicBehavior_SRC_carcinoma (Signet ring cell carcinoma histology), and ExtensionCS_200 (Tumor extension into muscularis propria).

With the dataset optimized, a multiple linear regression model was constructed. The model demonstrated excellent performance, achieving an R^2^ value of 0.95 and MSE of 0.05, as shown in the actual vs. predicted plot (Figure 8a). The importance score of each feature in the regression model was determined based on their correlation coefficients (Figure 8b). The top three variables with the highest positive scores were identified and plotted against the 5-year survival rate, revealing a strong positive relationship (Figure 8c). Conversely, the top three variables with the highest negative scores were plotted, illustrating a marked negative relationship with the 5-year survival rate (Figure 8d). These visualizations highlight the critical influence of these variables on patient outcomes, showcasing both positive and negative factors that significantly correlate with the 5-year survival rates.

## 4. Discussion

Esophageal cancer (EC) remains a significant and critical area of focus in medical research due to its severe prognosis and high mortality rates. Understanding the 5-year survival rate is crucial for assessing the long-term outcomes of EC patients, providing essential insights into the effectiveness of treatments and interventions [19]. Various approaches have been developed to predict prognosis factors, including nomograms and image-based models, each offering valuable predictive capabilities. However, these methods have limitations. Nomograms often rely on a limited number of clinical variables, missing the complexity of patient data. In contrast, image-based models require high-quality imaging and sophisticated analysis techniques, which may not be available in all clinical settings [7,20]. Herein, we introduce an ML approach for both the prediction and interpretation of factors influencing 5-year survival, utilizing a freely available database.

The EC dataset was downloaded from the SEER database, with a comprehensive selection of predictor variables, including demographic, clinicopathological, and treatment data. Following data preprocessing, exploratory data analysis revealed that the dataset is imbalanced and exhibits homogeneity. These characteristics rendered unsupervised learning algorithms, PCA and hierarchical clustering analysis ineffective [21]. Consequently, supervised learning approaches were recommended to achieve more accurate and reliable predictions.

Among supervised learning algorithms tested, RF achieved the highest sum score in the test set. Therefore, feature importance scores were extracted from the RF model, identifying the most influential variables in predicting 5-year survival. Our results align with several studies, indicating that surgery, summary stage, tumor size, metastasis, AJCC stage, and age are significant predictors of EC prognosis [22,23,24]. Notably, other reports have shown that the prognostic impact of these variables is not uniform across all settings; for example, age appears to worsen survival mainly in older patients in some cohorts [25], while AJCC stage alone provides only moderate discrimination of survival, particularly in intermediate stages [26]. These findings suggest that the prognostic value of individual variables should be interpreted with caution, as it may vary across cohorts and clinical settings. In contrast, the ML approach may help address this limitation by estimating prognosis from the combined contribution of multiple variables rather than relying on any single factor alone. In terms of ML model performance, our model yielded results comparable to a study using ML (XGBoost) to predict 5-year survival of EC patients from the SEER database (ROC-AUC 0.84 vs. 0.85) [11]. However, our ML model construction included data splitting for validation on the test set, providing a more robust assessment of model performance. The previous study also identified surgery, age, and AJCC stage as the main contributing variables for classification, which is consistent with our findings. Both studies imply the potential of ML approaches in identifying prognosis indicators for EC. This reinforces the value of integrating ML techniques to enhance prognostic assessments and support clinical decision-making in EC management.

We then integrated ML with statistical analysis, including Cox regression and KM analysis, to understand the relationship between the key predictors and outcome variables. The results from Cox and KM analyses were consistent with the ML findings, confirming that all the identified categories in each key predictor are statistically significant in predicting survival. Our results pinpoint that surgery is the most influential variable on survival prognosis. This finding aligns with previous studies indicating that surgery is one of the most important factors influencing patient survival. EC patients who undergo surgery have approximately a 30–45% higher chance of surviving another 5 years or longer compared to those without surgery [27]. Based on our analysis, surgical intervention may be associated with improved survival in EC patients and may represent a potentially important factor in treatment planning.

Other influential predictors of survival in our analysis included tumor spread or lymph node involvement, tumor size, and age. Although these are established prognostic markers in EC [28], our findings particularly highlight the adverse prognostic impact of metastatic burden, especially AJCC M1b. Patients with adverse features such as no surgery, distant site/node involvement, tumor size > 50 mm, and age ≥ 64 years had median survival ranging from 13 to 8 months, compared with the overall median survival of 15 months in the cohort. The poorest outcome was observed in patients with AJCC M1b, whose median survival was 6 months. Pairwise analysis further showed that patients with AJCC M1b, either alone or in combination with other adverse variables, consistently had the poorest survival (6 months) (see Supplementary Materials). Together, these findings suggest that distant metastatic spread is a major determinant of prognosis. This is consistent with previous reports showing a median survival of approximately 5 months for M1b disease [29]. The AJCC 8th edition no longer subdivides distant metastasis into M1a and M1b. In the updated system, celiac and cervical lymph nodes are classified as regional rather than distant disease, and metastatic status is simplified to M0 or M1. This revision was introduced because patients previously classified as M1a were found to have survival outcomes more similar to stage III disease than to true stage IV disease [30].

An exploratory temporal analysis was conducted in response to the observed linear increase in survival rates from 2004 to 2015. This trend prompted the use of linear regression as an exploratory tool to examine factors potentially associated with the observed pattern. A time-series dataset was then constructed and adjusted to eliminate multicollinearity. Our regression analysis suggested several variables that may be associated with favorable survival trends over time, including metastasis status, race, and grade. The absence of metastasis at diagnosis (MetsAtDXCS_0) was identified as a significant positive factor, consistent with its established prognostic relevance in previous studies [31]. Next, the Race_other category was composed predominantly of Asian/Pacific Islander patients (85%), which may have contributed to the observed association. This finding is broadly consistent with some previous reports suggesting more favorable survival outcomes among individuals of Asian descent [32]. However, because this category was heterogeneous and included multiple subgroups, the result should be interpreted with caution. The present analysis does not allow identification of the specific subgroup driving this trend, and residual confounding cannot be excluded. Subsequently, the Grade_unknown category was excluded from further discussion due to insufficient information on grading.

The model also identified negative influencing factors, including histology and tumor extension. EC with signet ring cell (SRC) carcinoma histology is more biologically aggressive and resistant to treatment than non-SRC types, thereby negatively impacting prognosis [33]. The category HistologicBehavior_Other was excluded from the discussion due to its nonspecific information. Furthermore, tumor extension into muscularis propria (ExtensionCS_200) typically indicates a more advanced and aggressive disease, which correlates with a poorer prognosis [34]. Overall, the model provides an exploratory view of factors potentially associated with patient survival over time. However, interpretation should be approached with caution, as some factors, such as grade and histology, contain unspecified information, which may limit the accuracy of the conclusions.

In our study, the clinical value of the ML approach lies in its ability to assess multiple interrelated clinicopathologic variables simultaneously within a complex dataset. Rather than replacing simpler established methods, ML was used as a complementary tool to identify important prognostic patterns, which were then interpreted alongside conventional statistical analyses. This combined approach provides a broader and more integrated understanding of prognostic factors in esophageal cancer.

The study acknowledges several limitations. Survival months were transformed into a binary 5-year survival outcome for classification modeling, which simplified the analysis but reduced the time-to-event information available from the original survival data. In addition, 14,582 cases were excluded due to missing data, representing 33.3% of the initial cohort. This substantial exclusion may have introduced selection bias and reduced the representativeness of the final study population. Furthermore, although internal validation was performed using a test set, the absence of external validation remains a significant limitation, potentially affecting the reliability of our results. The SEER database, which serves as the source of our data, presents additional challenges. Several variables within the SEER database are temporally constrained. For example, the AJCC (6th edition) and Collaborative Stage (CS) variables are only available for the period from 2004 to 2015. After 2015, the staging system transitioned to different criteria, such as the AJCC (8th edition), which has been in use since 2018. These changes introduce complexities and inconsistencies when analyzing data across different time periods, making it difficult to compare results over time. To address this, we restricted our data collection to the period from 2004 to 2015, ensuring data consistency used in the analysis. Notably, the AJCC 8th edition provides a more contemporary staging framework for esophageal cancer. In the TNM staging system, later editions use a more granular nodal classification and a simplified distant metastasis classification [23,30]; therefore, stage-related findings based on the AJCC 6th edition should be interpreted with caution in the context of current clinical practice. Another issue with SEER data is the presence of unspecified categories, such as “No/Unknown,” which poses challenges not only in model performance but also in the interpretation of results. These unspecified categories can obscure the true nature of the data and reduce the clarity of the findings, further complicating the analysis.

Regarding the limitations of the temporal analysis, the time-series dataset was created by aggregating patient-level data into yearly summaries, with continuous variables expressed as annual medians and categorical variables as yearly percentages. Although this approach allowed broad temporal patterns to be explored, it reduced patient-level variability and may have obscured clinically meaningful correlations. In addition, converting categorical variables into percentages introduced substantial multicollinearity, because an increase in one category within the same variable corresponded to a decrease in another. To address this issue, we applied feature selection methods and reduced the number of variables to 12. Although this reduction did not markedly affect model performance, it may have compromised interpretability by excluding variables potentially associated with the outcome. Therefore, caution is warranted when interpreting these results. Additionally, the dataset was limited to just 12 rows, each representing a single year from 2004 to 2015, due to the lack of finer time granularity (e.g., days or months) in the SEER database. This constraint confined us to using simpler algorithms like linear regression, which primarily identifies linear correlations and lacks the ability to recognize more complex patterns. It also restricted the application of more robust modeling steps, such as data splitting and cross-validation, ultimately impacting the overall reliability of the model.

## 5. Conclusions

This study demonstrates the effectiveness of using machine learning on the freely available SEER database to identify significant prognostic factors for EC. A multi-step approach was employed to predict 5-year survival, with RF emerging as the top-performing algorithm. Feature importance was extracted from the RF model, highlighting key contributors to data discrimination. Subsequent statistical analysis confirmed that variables such as surgery, summary stage, and tumor size are critical prognostic factors. Additionally, the exploratory temporal analysis provided a dynamic perspective on survival trends over time, revealing both positive and negative factors that significantly influence these trends. Our linear regression model identified crucial contributors, such as the positive impact of non-metastasis at diagnosis and the negative influence of the aggressive SRC histological subtype. Despite the study’s contributions, limitations such as the lack of external validation and challenges inherent in temporal analysis suggest that future research should focus not only on improving model performance but also on enhancing its reliability. Overall, this study underscores the potential of machine learning to transform the prognosis and management of EC, paving the way for more personalized and effective treatment strategies.

## Figures and Tables

**Table 1:** Summary of data characteristics.

Characteristics	SEER Variable	Value/Category ^c^	Training Set (*n* = 9727)	Test Set (*n* = 4169)	*p*-Value
Predictor variables: Demographic data					
Age ^a^	“Age recode with single ages and 90+”	[23, 80]	64	64	≥0.01
	[Rename: “Age”]				
Sex	“Sex”	Male	7863 (80.84%)	3354 80.45%)	≥0.01
		Female	1864 (19.16%)	815 (19.55%)	
Race	“Race recode (W, B, AI, API)”	White	8180 (84.10%)	3478 (83.43%)	≥0.01
	[Rename: “Race”]	Other	1547 (15.90%)	691 (16.57%)	
Origin	“Origin recode NHIA (Hispanic, Non-Hisp)”	Hispanic	724 (7.44%)	302 (7.24%)	≥0.01
	[Rename: “Hispanic”]	Non-Hispanic	9003 (92.56%)	3867 (92.76%)	
Marital status	“Marital status at diagnosis”	Married	5790 (59.53%)	2475 (59.37%)	≥0.01
	[Rename: “MaritalStatus”]	Other	3937 (40.47%)	1694 (40.63%)	
Income	“Median household income inflation adj to 2022”	Medium	6862 (70.55%)	2944 (70.62%)	≥0.01
	[Rename: “Income”]	Other	2865 (29.45%)	1225 (29.38%)	
Predictor variables: Clinicopathological data					
Histology	“ICD-O-3 Hist/behav, malignant”	Adenocarcinoma	5477 (56.31%)	2327 (55.82%)	≥0.01
	[Rename: HistologicalBehavior]	Other	4250 (43.69%)	1842 (44.19%)	
Site	“Primary Site—labeled”	C15.5	6201 (63.75%)	2672 (64.09%)	≥0.01
	[Rename: “PrimarySite_code”]	Other	3526 (36.25%)	1497 (35.91%)	
Sequence	“Sequence number”	Primary	8212 (84.42%)	3502 (84.00%)	≥0.01
	[Rename: “SequenceNumber”]	Other	1515 (15.58%)	667 (16.00%)	
Grade	“Grade Recode (thru 2017)”	Grade III	4155 (42.72%)	1807 (43.34%)	≥0.01
	[Rename: “Grade”]	Other	5572 (57.28%)	2362 (56.66%)	
AJCC T stage	“Derived AJCC T, 6th ed (2004–2015)”	T3	4235 (43.54%)	1790 (42.94%)	≥0.01
	[Rename: “AJCC_T”]	Other	5492 (56.46%)	2379 (57.06%)	
AJCC N stage	“Derived AJCC N, 6th ed (2004–2015)”	N1	5671 (58.30%)	2391 (57.35%)	≥0.01
	[Rename: “AJCC_N”]	N0	4056 (41.70%)	1778 (42.65%)	
AJCC M stage	“Derived AJCC M, 6th ed (2004–2015)”	M0	6896 (70.90%)	2983 (71.55%)	≥0.01
	[Rename: “AJCC_M”]	Other	2831 (29.10%)	1186 (28.45%)	
Summary stage	“SEER Combined Summary Stage 2000 (2004–2017)”	Dist+LN ^d^	3316 (34.09%)	1430 (34.30%)	≥0.01
	[Rename: “SummaryStage”]	Other	6411 (65.91%)	2739 (65.69%)	
Tumor extension	“CS extension (2004–2015)”	Localized	3042 (31.27%)	1302 (31.24%)	≥0.01
	[Rename: “ExtensionCS”]	Other	6685 (68.73%)	2866 (68.76%)	
Lymph node involvement	“CS lymph nodes (2004–2015)”	Not involved	3769 (38.75%)	1667 (39.99%)	≥0.01
	[Rename: “LymphNodesCS”]	Other	5958 (61.25%)	2502 (60.01%)	
Metastasis at diagnosis	“CS mets at dx (2004–2015)”	No metastasis	7189 (73.91%)	3096 (74.26%)	≥0.01
	[Rename: MetsAtDXCS]	Other	2538 (26.09%)	1073 (25.74%)	
Tumor size ^a^	“CS tumor size (2004–2015)”	[0, 990]	50	40	≥0.01
	[Rename: “TumorSizeCS”]				
Familial polyposis ^b^	“CS tumor size (2004–2015)”	Yes	331 (3.40%)	177 (4.25%)	≥0.01
	[Rename: “TumorSize_CS_polyposis”]	No	9396 (96.60%)	3992 (95.75%)	
Number of malignant tumor ^a^	“Total number of in situ/malignant tumors for patient”	[1, 8]	1	1	≥0.01
	[Rename: “InSituMalignantTumorsTotal”]				
Number of benign tumor ^a^	“Total number of benign/borderline tumors for patient”	[0, 3]	0	0	≥0.01
	[Rename: “BenignTumorsTotal”]				
Predictor variables: Treatment data					
Surgery	“RX Summ–Surg Prim Site (1998+)”	No surgery	5784 (59.46%)	2492 (59.77%)	≥0.01
	[Rename: “SurgicalTreatment”]	Other	3943 (40.54%)	1677 (40.24%)	
Lymph node removal	“RX Summ–Scope Reg LN Sur (2003+)”	Yes	3888 (39.97%)	1644 (39.43%)	≥0.01
	[Rename: “ScopeRegionalLNSurgery”]	No/Unknown	5839 (60.03%)	2525 (60.57%)	
Radiation	“Radiation recode”	Yes	6505 (66.88%)	2769 (66.42%)	≥0.01
	[Rename: “Radiation”]	No/Unknown	3222 (33.12%)	1400 (33.58%)	
Sequence of surgical radiation	“RX Summ--Surg/Rad Seq”	Radiation before	1985 (20.41%)	811 (19.45%)	≥0.01
	[Rename: “SurgicalRadiationSequence”]	Other	7742 (79.59%)	3358 (80.55%)	
Chemotherapy	“Chemotherapy recode (yes, no/unk)”	Yes	7141 (73.41%)	2955 (70.88%)	<0.01
	[Rename: “Chemotherapy”]	No/Unknown	2586 (26.59%)	1214 (29.12%)	
Outcome variable					
5-year survival	“Survival months”	Yes	2101 (21.60%)	900 (21.59%)	≥0.01
	[Rename: “Y5_Survival”]	No	7626 (78.40%)	3269 (78.41%)	

^a^ Continuous variable. ^b^ Familial polyposis variable derived from “CS tumor size (2004–2015): code 998”. ^c^ Min–Max for continuous variables, and an important category for categorical variables. ^d^ Refers to code 7 (Distant site(s)/node(s) involved).

**Table 2:** Model performance on the test set.

Algorithm ^a^	Accuracy	ROC-AUC	F1 Score	Sum
RF	0.78	0.84	0.58	2.20
ANN	0.78	0.82	0.56	2.18
KNN	0.73	0.76	0.51	2.00
AdaBoost	0.80	0.77	0.50	2.08
Naive Bayes	0.63	0.74	0.47	1.85

ROC-AUC: Area under the ROC (Receiver Operating Characteristic) curve. ^a^ RF: Random Forest; ANN: Artificial Neural Network; KNN: K-Nearest Neighbors.

## Data Availability

The datasets generated and/or analysed during the current study are openly available in the Github repository, https://github.com/Piman-Pocasap/EC_SEER_ML_analysis.git (accessed on 11 April 2026).

## Supplementary Materials

The following supporting information can be downloaded at: https://www.mdpi.com/article/10.3390/jcm15083049/s1. Figure S1. Confusion matrices of the machine learning models. Figure S2. Pairwise median survival heatmap for key predictors.
