# Supplementary material for: Identification of Prognostic Factors in Esophageal Cancer Using Machine Learning: A Retrospective Study Based on the SEER Database
Source: J Clin Med. 2026 Apr 16;15(8):3049. doi: 10.3390/jcm15083049 (PMC13116311; doi:10.3390/jcm15083049)
Supplement: Supplementary file 1 [file jcm-15-03049-s001.zip › jcm-4222984-supplementary.pdf]

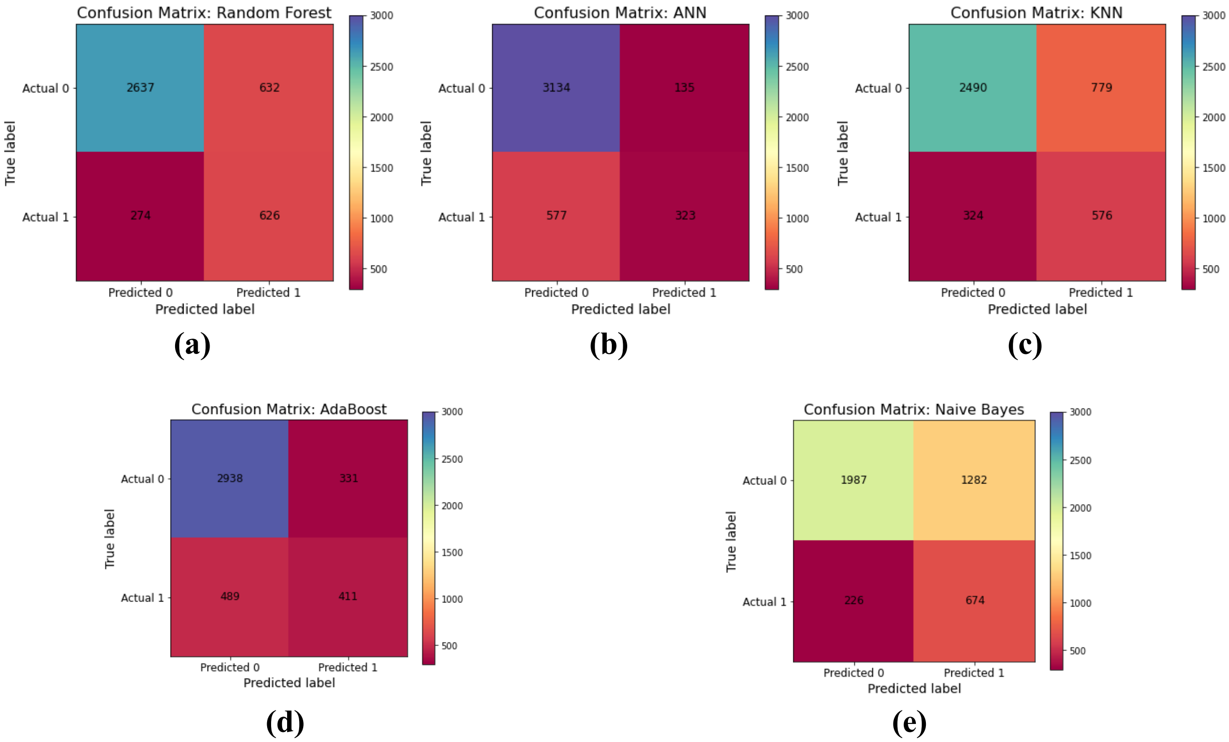

Figure S1. Confusion matrices of the machine learning models: (a) Random Forest (RF), (b) artificial neural network (ANN), (c) k-nearest neighbors (KNN), (d) AdaBoost, and (e) Naïve Bayes.

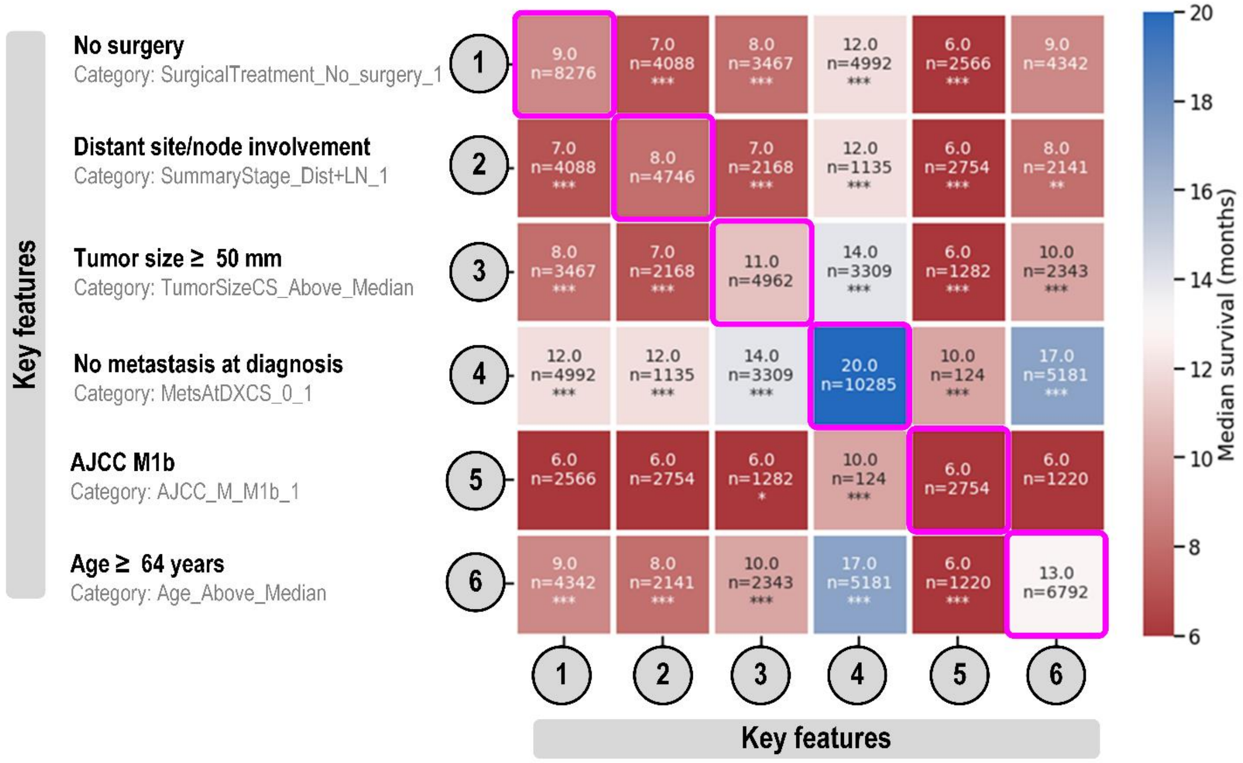

Figure S2. Pairwise median survival heatmap for key predictors. A heatmap summarizes median survival time (months) for patients meeting the joint condition of the key predictors. Diagonal cells represent the median survival for patients with the corresponding single predictor present. Cell annotations show the median survival and subgroup size (*n*). Where indicated, significance markers denote Mann–Whitney U tests comparing each off-diagonal subgroup to the corresponding row baseline ($p < 0.01$).
